# Efficacy and safety of Buzhong Yiqi Decoction in improving cancer-related fatigue and immunity of cervical carcinoma patients: A protocol of randomized controlled trial

**DOI:** 10.1097/MD.0000000000027938

**Published:** 2021-12-10

**Authors:** Juan Hu, Xia Li, Yanping Fang, Jin Peng

**Affiliations:** a The Hospital Affiliated to Jianghan University (Wuhan No. 6 Hospital), Wuhan, Hubei Province, China; b The First People's Hospital of Wuhan Jiangxia District, Wuhan, Hubei Province, China.

**Keywords:** Buzhong Yiqi Decoction, cancer related fatigue, cervical carcinoma, Chinese medicine, RCT

## Abstract

**Background:**

Cancer-related fatigue (CRF) is essentially universal in cervical carcinoma patients. It develops rapidly, with physical and mental manifestations including generalized weakness, diminished concentration or attention, and it has a negative impact in overall quality of life. Buzhong Yiqi Decoction (BYD), a classical Chinese medical prescription, could be used for allergic rhinitis, gut microbiota disorders, and chronic obstructive pulmonary disease. We preliminarily found that BYD could relieve CRF in cervical carcinoma patients. However, there are few trials on whether BYD could relieve CRF and improve immunity in cervical carcinoma patients.

**Methods:**

This is a double-blinded, randomized, controlled clinical trial. From December 1, 2021 to May 31, 2022, cervical carcinoma patients with CRF will be assessed for randomization into treatment group (BYD) and control group (BYD simulation) in a 1:1 ratio. The outcomes are cancer fatigue scale, self-rating anxiety scales, self-rating depression scales, Pittsburgh sleep quality index, and immunity index (CD3^+^, CD4^+^, and CD8^+^) before and after the treatment. Statistical analysis will be performed using SPSS v22.0 software.

**Results and conclusions:**

The study will clarify the efficacy and safety of BYD in improving CRF and immunity in cervical carcinoma patients.

**Trial registration:**

OSF Registration number: DOI 10.17605/OSF.IO/QFNMD.

## 1 Introduction

Cervical carcinoma, a malignant tumor that develops in the cervix, is mainly related to long-term infection. It is a common malignant tumor all over the world, with a high incidence, which has seriously affected women's physical and mental health.^[1,2]^ In recent years, chemotherapy has gradually become one of prior treatment methods for cervical carcinoma.^[3–5]^ It has been reported that low-dose chemotherapeutics could increase the sensitivity of radiotherapy in cervical carcinoma patients. With the continuous development of concurrent radiotherapy and chemotherapy, the efficacy and local control rate of radiotherapy have gradually increased, and the recurrence rate has initially decreased. Also, a large number of clinical studies have shown that concurrent radiotherapy and chemotherapy can significantly improve the survival rate of patients with advanced cervical carcinoma.^[6,7]^

Fatigue is one of the most common side effects of malignant tumor and its treatment, known as cancer related fatigue (CRF). It's a subjective experience self-reported symptoms.^[8,9]^ In recent years, CRF has gradually received clinical attention and intervention. CRF is widespread in cancer patients, and it is even a more common side effect in patients receiving chemotherapy and radiotherapy. It's estimated that the prevalence ranges from 25% to 99% in cancer patients after treatment.^[10,11]^ In cervical carcinoma patients, CRF was reported by 23% of long-term survivors at a mean of 11 years after treatment, and 28% in patients with chemoradiation.^[12]^ CRF develops rapidly, with physical and mental manifestations including generalized weakness, diminished concentration or attention, and it has a negative impact in overall quality of life.

A variety of interventions have been used for CRF, including physical activity, psychosocial and pharmacological interventions, yet with limited or unclear feasibility and efficacy.^[12]^ Traditional Chinese medicine (TCM) could control the development of cervical carcinoma. As an adjuvant treatment, it could also reduce adverse reactions and side effects of radiotherapy and chemotherapy, improve the long-term curative effect and the quality of life. Buzhong Yiqi Decoction (BYD), a traditional Chinese prescription, is composed of Huangqi (*Astragalus membranaceus*), Baizhu (*Atractylodes atractylodes*), Chenpi (*Pericarpium citri reticulatae*), Shengma (*Rhizoma cimicifugae*), Chaihu (*Radix bupleuri*), Rensheng (*Ginseng*), Gancao (*Liquorice*), and Danggui (*Radix Angelicae Sinensis*). Clinically, BYD could be used for allergic rhinitis, gut microbiota disorders, and chronic obstructive pulmonary disease.^[13–15]^ We preliminarily found that BYD could be used for CRF in cervical carcinoma patients. However, few trials have been reported on whether BYD could reduce CRF in cervical carcinoma patients. In this study, we try to conduct a randomized controlled trial to clarify the efficacy and safety of BYD in relieving CRF and improving immunity in cervical carcinoma patients.

## 2 Methods

### 2.1 Trial design

**Figure 1:**
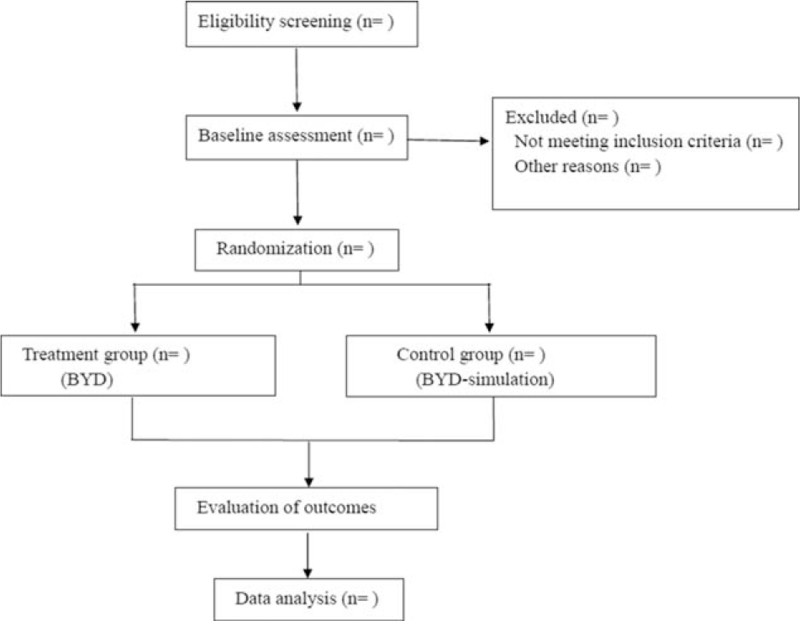
Flow diagram.

This is a randomized, controlled clinical trial, and it has been approved by the Ethics Committee of the Hospital Affiliated to Jianghan University. The study will be carried out in accordance with the Declaration of Helsinki. It conforms to the SPIRIT 2013 Statement, and the results will be reported according to the CONSORT Statement extension for trials (Fig. 1). This study protocol has been registered with Open science framework Registry platform (OSF Registration number: DOI 10.17605/OSF.IO/QFNMD).

### 2.2 Participants

#### 2.2.1 Recruitment

From December 1, 2021 to May 31, 2022, cervical carcinoma patients diagnosed with CRF will be assessed for eligibility. Before randomization, all patients will sign a written informed consent, and they can freely choose whether to discontinue the trial at any time.

#### 2.2.2 Inclusion and exclusion criteria

Patients will be included if they meet the following criteria:

1) primary diagnosis of cervical carcinoma in stage Ib–IVa;
2) complained of fatigue and a diagnosis with CRF;
3) aging from 18 to 70;
4) with signed informed consent.

Patients will be excluded if they are:

1) complicated with other malignant tumors;
2) received surgery before;
3) with severe abnormal liver or kidney function;
4) history of allergy to BYD.

### 2.3 Randomization

For allocation, patients will be simply assigned into treatment group (BYD) and control group (BYD simulation) in a 1:1 ratio. The randomization sequence will be generated by a statistician uninvolved in the statistical analysis. The random numbers will be placed in opaque, sealed envelopes and kept in a safe place. Each participant had a 50% probability of receiving each intervention upon selection.

### 2.4 Blinding

This is a double blinded, and dummied study. The physician, patients, and assessors will all be blinded to the allocation. To maintain blindness, the decoction and simulations will be provided as granules. The BYD-simulations are in the same appearance, package, color, taste, and administration with BYD.

### 2.5 Interventions

Patients diagnosed with CRF in the treatment group will be treated with traditional Chinese prescription of BYD as granules, including Huangqi (*A membranaceus*) 15 g, Baizhu (*A atractylodes*) 10 g, Chenpi (*P citri reticulatae*) 10 g, Shengma (*R cimicifugae*) 6 g, Chaihu (*R bupleuri*) 12 g, Dangshen (*Radix codcnopsitis pilosulas*) 15 g, Gancao (*Liquorice*) 15 g, and Danggui (*R Angelicae Sinensis*) 6 g. The granules will be provided by the Beijing Tcmages Pharmaceutical Co., Ltd., and they will be orally taken by dissolving in 50 mL warm water, twice a day, for 14 days. While in the control group, BYD simulations made of amylum will be applied for patients with the same administration method.

### 2.6 Outcome variables

The primary outcome is the change of fatigue scores measured by cancer fatigue scale before and after the treatment. The secondary outcomes are self-rating anxiety scales,^[16]^ self-rating depression scales,^[17]^ Pittsburgh sleep quality index,^[18]^ and immunity index (CD3^+^, CD4^+^, and CD8^+^) before and after the treatment. Blood routine, liver and kidney function, coagulation, and adverse events will also be recorded.

### 2.7 Sample size

In our preliminary study, the change of cancer fatigue scale scores in the treatment group was 6.65 ± 2.12, while 3.5 ± 1.53 in the control group. Considering a 2-sided *t* test, with 5% type I error at 80% power, and a potential dropout of 10%, the required total sample size will be 110.

### 2.8 Statistical analysis

Statistical analysis will be performed using SPSS v22.0 software. The measurement data is expressed as mean ± standard deviation, and Student *t* test, Wilcoxon rank-sum test, or Manne–Whitney *U* test after normality and homogeneity test. The count data is expressed as percentage (%) and *χ*^2^ test will be applied. *P* < .05 is considered statistically significant.

## 3 Discussions

Cancer-related fatigue (CRF) is essentially universal in patients receiving cancer treatment other than surgery, and it can be influenced by many factors, such as medications, pain, sleepless, and depression, etc^[19,20]^ It develops rapidly, and patients may be complained with generalized weakness, diminished concentration or attention. At present, the understanding of the etiology and pathogenesis of CRF is still limited, and there are only a few limited or unclear treatment methods. Therefore, a treatment with high practicability, safe and effective treatment needs to be further studied.

As an adjuvant treatment, TCM could reduce adverse effects to improve the quality of life. BYD, a traditional Chinese prescription, could regulate immune function, and act as an effective biological regulator to promote protein synthesis and energy metabolism. Clinically, BYD could be used for allergic rhinitis, gut microbiota disorders, and chronic obstructive pulmonary disease. The symptoms of CRF are similar to Qi deficiency in TCM, which can be treated by BYD. Therefore, in this study, we try to conduct a randomized controlled trial to clarify the efficacy and safety of BYD in relieving CRF and improving immunity in cervical carcinoma patients.

Yet, it has to be claimed some limitations in this protocol. First, several outcomes are measured by subjective scale, and there might be some certain biases. Second, the recruitment of patients is single and limited in Wuhan, which may affect the conclusions to some extent.

## Author contributions

**Conceptualization:** Jin Peng.

**Data collection:** Juan Hu and Xia Li.

**Funding acquisition:** Jin Peng.

**Investigation:** Juan Hu.

**Protocol design:** Juan Hu and Xia Li.

**Resources:** Xia Li.

**Software operating:** Xia Li and Yanping Fang.

**Supervision:** Yanping Fang and Jin Peng.

**Writing – original draft:** Juan Hu and Xia Li.

**Writing – review and editing:** Juan Hu and Jin Peng.
